# CS Ratio is an immune-related prognostic biomarker for cervical cancer

**DOI:** 10.3389/fonc.2025.1547529

**Published:** 2025-08-27

**Authors:** Peiqin Shi, Wenwen Zhang, Qingqing Yao, Zhanna Yuan, Jiaqi Wang, Ziye Yang, Pengpeng Qu

**Affiliations:** ^1^ Clinical School of Obstetrics and Gynecology Center, Tianjin Medical University, Tianjin, China; ^2^ Tianjin Institute of Gynecology Obstetrics, Tianjin Central Hospital of Gynecology Obstetrics, Tianjin, China; ^3^ Department of Gynecological Oncology, Tianjin Central Hospital of Gynecology Obstetrics, School of Medicine, Nankai University, Tianjin, China; ^4^ Department of Obstetrics and Gynecology, Tianjin Medical University Baodi Hospital, Tianjin, China; ^5^ Department of Gynecological Oncology, Tianjin Central Hospital of Gynecology Obstetrics, Tianjin, China

**Keywords:** SPP1, CXCL9, cervical cancer, TCGA, GEO

## Abstract

**Background:**

The tumor microenvironment (TME) plays a crucial role in cancer progression but its complex structure significant variability among patients present considerable challenges for research. Recent studies have demonstrated that macrophage polarization states defined by the expression levels of CXCL9 SPP1 (CS Ratio) are more prognostically relevant than traditional M1/M2 markers. The CS polarization state reflects a highly coordinated network of pro-tumor anti-tumor variables offering a simplified yet effective immune response indicator for the complex TME. The CS Ratio has been shown to correlate with the abundance of anti-tumor immune cells the gene expression programs of tumor-infiltrating cells responses to immunotherapy. Cervical cancer, one of the most common gynecological malignancies, still faces limited therapeutic options. CXCL9, a member of the CXC chemokine family, plays a critical role in immune regulation, inflammation, tumor growth, angiogenesis, and metastasis. Similarly, SPP1, a cytokine, influences immune-related pathways by regulating molecules such as interferon-γ and interleukin-12. However, no studies have systematically investigated the role of the CS Ratio in cervical cancer or its relationship with immunotherapy characteristics. Research in this area could provide critical insights into the role and clinical potential of the CS Ratio in cervical cancer and related tumors.

**Methods:**

The expression ratio of CXCL9 to SPP1 was analyzed in cervical cancer patients using data from the Gene Expression Omnibus (GEO) database, which revealed significant differences. Data for cervical cancer patients were obtained from The Cancer Genome Atlas (TCGA) database. The optimal cutoff value for the CS Ratio was determined using the maxstat package in R, and Kaplan-Meier (KM) survival curves were constructed. Patients were categorized into High and Low groups based on the median CS Ratio. Immune scores were analyzed, and immune cell infiltration was assessed using CIBERSORT. Differences in the CS Ratio were evaluated across patients with varying pathological T stages and FIGO stages. Additionally, receiver operating characteristic (ROC) analysis was performed using the pROC package in R to calculate the area under the curve (AUC). Univariate and multivariate Cox regression analyses were performed to evaluate the potential of the CS Ratio as an independent prognostic factor in cervical cancer. A Cox regression-based nomogram integrating four key features was subsequently developed for the TCGA-CESC cohort. Nomogram performance was assessed using calibration curves and ROC analysis.

**Results:**

The CS Ratio was significantly lower in cervical cancer patients compared to normal controls (P < 0.05). KM survival curves indicated that patients in the CS High group exhibited better prognoses. Immune score analysis revealed significantly higher immune scores (P < 0.05) and lower tumor purity (P < 0.05)in the CS High group compared to the Low group. CIBERSORT analysis revealed significantly higher proportions of CD8+ T cells (P < 0.05) and M1 macrophages (P < 0.05), and a significantly lower proportion of M2 macrophages (P < 0.05), in the CS High group compared to the Low group. The CS Ratio significantly decreased with advancing FIGO stage (P < 0.05). Both univariate (P < 0.05) and multivariate Cox regression analyses (P < 0.05) confirmed the CS Ratio as an independent prognostic factor. ROC analysis demonstrated that the CS Ratio had higher AUC values for predicting 1-year (AUC=0.69), 3-year (AUC=0.66), and 5-year OS (AUC=0.68) than CXCL9 or SPP1 alone. The Cox regression-based nomogram integrating four key features demonstrated predictive capability for 1-, 3-, and 5-year OS in CESC patients (Concordance Index = 0.751; 95% CI: 0.678–0.824; p = 1.50Í10-11). Significant survival differences were observed between the high-risk and low-risk groups based on the nomogram score. ROC analysis yielded high AUC values for survival prediction: 0.85 (95% CI: 0.94-0.75) at 1-year, 0.74 (95% CI:0.84-0.64) at 3-year, and 0.72 (95% CI:0.84-0.61) at 5-year.

**Conclusion:**

The CS Ratio may serve as a more effective prognostic biomarker for cervical cancer patients.

## 1 Introduction

Cervical cancer remains the fourth most common cancer among women, with 527,624 new cases and 265,672 deaths reported in 2018 (1). Persistent human papillomavirus (HPV) infection is a primary risk factor for cervical cancer (2). Advances in bioinformatics technology have created opportunities to identify biomarkers that enhance the diagnosis and management of cervical cancer.

Chemokines, a class of signaling cytokines, play a crucial role in the interaction between tumor cells and their microenvironment. Through receptor interactions, chemokines regulate immune infiltration, tumor-associated angiogenesis, host immune responses, and tumor cell proliferation (3, 4). Interferon-gamma (CXCL9), a member of the CXC chemokine family, attracts CXCR3-expressing (CXCR3-a and CXCR3-b) T lymphocytes and is involved in various physiological and pathological processes. The CXCL9-CXCR3 signaling pathway is critical for immune cell migration, differentiation, and activation (5, 6). Overexpression of CXCL9 has been linked to increased T-cell infiltration and improved overall survival (OS) in ovarian cancer (7). Chow et al. demonstrated that CXCL9, produced by CD103+ dendritic cells alongside CD8+ T cells (CXCR3+), is vital for the efficacy of anti-PD-1 therapy in models of melanoma, colon adenocarcinoma, and breast adenocarcinoma (6). Ester et al. showed that HPV E6/E7-induced LIF (via NFκB) suppresses CXCL9 in tumor-associated Macrophages (TAMs) and type I IFN in pDCs, highlighting CXCL9 as a functional biomarker—its reactivation upon LIF blockade recruits CD8+ T cells and sensitizes tumors to immune checkpoint inhibition (ICI) (8). SPP1, a member of the small integrin-binding ligand N-linked glycoprotein (SIBLING) family, also known as osteopontin-like protein or early T-lymphocyte activation 1 protein, specifically binds and activates matrix metalloproteinases (MMPs) in cancers (9). Its biological functions predominantly involve immune responses, biomineralization, and tissue remodeling. SPP1 is also implicated in cell growth, proliferation, migration, apoptosis, and chemotaxis. Previous studies have indicated that SPP1 is overexpressed in various cancers and can serve as a predictor of poor outcomes, including in ovarian cancer (10), glioblastoma (11), hepatocellular carcinoma (12), and gastric cancer (13). Kaidi et al. demonstrated through comprehensive bioinformatics analysis that SPP1 may serve as a promising prognostic biomarker for patients with cervical cancer (14). CXCL9 and SPP1 already have established and arguably opposing roles in cancer biology (15–17). However, the expression patterns, prognostic significance, and underlying molecular mechanisms of CXCL9 and SPP1 in cervical cancer remain unclear. Therefore, this study aims to explore the expression profiles of CXCL9 and SPP1 in cervical cancer tissues and assess their potential clinical value.

Growing evidence underscores the critical role of the TME in tumor progression. TAMs within the TME play a pivotal role in tumorigenesis and progression by promoting invasion, migration, angiogenesis, and suppressing anti-tumor immunity (18, 19). Tumor-associated macrophages (TAMs) exhibit dual phenotypes: M1-like (anti-tumor, pro-inflammatory) and M2-like (pro-tumor, immunosuppressive), with the latter predominating in tumors to promote angiogenesis, metastasis, and immune evasion (20, 21). Recent studies have demonstrated that the expression of CXCL9 and SPP1 (CS) exhibits a strong prognostic association, surpassing traditional M1 and M2 markers, as revealed by single-cell analysis in head and neck squamous cell carcinoma. The ratio of CXCL9 to SPP1 (CS Ratio) has been closely linked to the prognosis of initial macrophages. Furthermore, the polarization of CS macrophages (defined by CXCL9 and SPP1 expression) unveils a highly coordinated network encompassing variables that promote or suppress tumor progression. Despite the complexity of the TME, it orchestrates consistent biological responses that govern human cancer progression, with CS macrophage polarization serving as a critical variable. The CS Ratio characterizes the abundance of anti-tumor immune cells, the gene expression programs of various tumor-infiltrating immune cell types, the communication networks governing tumor control or progression, and the responsiveness to immunotherapy (22). High-risk HPV infection is widely recognized as the etiological factor in nearly all cervical cancers and certain forms of head and neck squamous cell carcinoma (2, 23, 24). The predominant histologic subtype of cervical cancer is squamous cell carcinoma (SCC). HPV serves as a primary etiological agent in both cervical SCC and head and neck SCC (HNSCC). Current research on cervical cancer has revealed distinct functional roles of CXCL9 and secreted SPP1 in tumor pathogenesis (8, 14). In our study, we identified that cervical cancer patients with a high CS Ratio (CS Ratio Hi) exhibited improved prognoses. This suggests that the CS Ratio might influence cancer progression through immune regulation, positioning it as a promising prognostic biomarker in cervical cancer.

This study analyzed cervical cancer RNA sequencing data sourced from TCGA and GEO databases. The CS Ratio was defined as the expression level of CXCL9 divided by the expression level of SPP1. We compared the expression differences of CXCL9 and SPP1 between normal cervical tissues and cervical cancer samples. Furthermore, we evaluated their expression in public databases, their correlation with cancer prognosis, immune scores, immune cell infiltration, disease staging, univariate and multivariate Cox analyses, and ROC analysis to explore the potential of the CS Ratio as a prognostic biomarker in cervical cancer.

Transcriptome sequencing data from the TCGA database revealed that the CS Ratio is a significant and independent prognostic biomarker for cervical cancer patients. By categorizing patients into High and Low CS Ratio groups based on the median CS Ratio, we found that patients in the CS Ratio Hi group had better prognoses, higher immune scores, and stronger correlations with T cells and M1 macrophages. Moreover, patients with a high CS Ratio exhibited significantly lower T-stage and overall stage classifications.

## 2 Materials and methods

### 2.1 RNA sequencing data collection and analysis

Expression data for CXCL9 and SPP1, along with clinical information from 304 cervical cancer tissues, were sourced from TCGA public database (https://portal.gdc.cancer.gov/). We selected samples from the TCGA database for the analysis of CXCL9 and SPP1 expression in tumor tissues, while the combined analysis of TCGA and Genotype Tissue Expression (GTEx) databases was used for the normal tissue samples. Cervical cancer microarray data were obtained from the GEO database (https://www.ncbi.nlm.nih.gov/geo), specifically GSE6791 (platform: GPL570) and GSE9750 (platform: GPL96).

### 2.2 Differential expression analysis and enrichment analysis

Differentially expressed genes (DEGs) between the CS Ratio High and Low groups were identified using the “limma” R package. A cutoff threshold of |log2-fold change (FC)| > 2 and adjusted p-value (FDR) < 0.05 was applied. Heat maps and volcano plots were generated to visualize results of the differential expression analysis.

We performed enrichment analysis on the identified DEGs.For functional enrichment analysis, gene sets were downloaded from the Molecular Signatures Database (https://www.gsea-msigdb.org/gsea/msigdb), including c5.go.mf.v7.4.symbols.gmt, c5.go.bp.v7.4.symbols.gmt, and c5.go.cc.v7.4.symbols.gmt subsets. These gene sets provided the background for mapping the DEGs, and enrichment analysis was performed using the R package clusterProfiler. P value of < 0.001 and a FDR of < 0.001 were considered statistically significant. Additionally, the latest gene annotations for KEGG pathways were acquired via the KEGG REST API. We performed the same background mapping and enrichment analysis using the clusterProfiler package, setting minimum and maximum gene set sizes to 5 and 5000, P value of < 0.001 and a FDR of < 0.001 were considered statistically significant.

GSEA software was obtained from the GSEA website (https://www.gsea-msigdb.org/gsea/index.jsp). Samples were divided into High and Low groups according to the median CS Ratio, and the c2.cp.kegg.v7.4.symbols.gmt subset was downloaded from the Molecular Signatures Database to evaluate relevant pathways and molecular mechanisms. This analysis was based on gene expression profiles and phenotypic grouping, with the minimum gene set size set to 5 and the maximum to 5000, involving 1000 permutations; P value of < 0.001 and a FDR of < 0.001 were considered statistically significant.

### 2.3 Immune cell infiltration characterization

Expression data for the CXCL9 and SPP1 genes in cervical cancer patients were extracted and transformed using the log2(x+1) method for each expression value. The ratio of the two genes was defined as the CS Ratio. Additionally, gene expression profiles from cervical cancer patients were obtained and mapped to Gene Symbols. We further employed the R package IOBR, specifically its deconvo_CIBERSORT function, to reassess the levels of various immune cell types in each tumor based on gene expression profiles. Specifically, the analysis included naive B cells, memory B cells, plasma cells, CD8+ T cells, naive CD4+ T cells, resting memory CD4+ T cells, activated memory CD4+ T cells, follicular helper T cells, regulatory T cells (Tregs), γδ T cells, resting natural killer (NK) cells, activated NK cells, monocytes, M0 macrophages, M1 macrophages, M2 macrophages, resting dendritic cells, activated dendritic cells, resting mast cells, activated mast cells, eosinophils, and neutrophils.

We extracted gene expression profiles from cervical cancer patients, mapped gene identifiers to official Gene Symbols, and recalculated immune infiltration scores (B cells, CD4+ T cells, CD8+ T cells, neutrophils, macrophages, and dendritic cells) using the TIMER algorithm implemented in the IOBR R package. Patients were stratified into two groups based on CS Ratio (high vs low) to examine the association between CS Ratio levels and infiltration patterns of these six immune cell subtypes. Furthermore, the R package ESTIMATE was utilized to calculate Stromal, Immune, and Estimate Scores for each patient in the tumors based on gene expression. Using TCGA data, the correlation between immune cell infiltration and the CS Ratio High and Low groups was evaluated using the ESTIMATE method.

Additionally, tumor purity data for each patient obtained from prior studies was integrated with gene expression data. The correlation between tumor purity and CS Ratio in cervical cancer patients was analyzed. Survival data were acquired from TCGA, and the optimal cut-off value for tumor purity was calculated using the maxstat R package, with the minimum group size set at >25% and maximum group size at <75%. Patients were stratified into high- and low-purity groups based on this cut-off. Prognostic differences between groups were analyzed using the survfit function from the survival R package, with significance assessed by the Cox method.

### 2.4 Enrichment analysis of differential gene sets in CS ratio HL groups

We performed Gene Ontology (GO) enrichment analysis and Kyoto Encyclopedia of Genes and Genomes (KEGG) pathway analysis using the ClusterProfiler package. Functional analyses focused on biological processes (BP), cellular components (CC), and molecular functions (MF). KEGG pathway analysis revealed biological pathways associated with the DEGs. The significance threshold for enrichment was set at p < 0.05. GSEA software was obtained from the GSEA website, and samples were divided into two groups based on the CS Ratio. The c2.cp.kegg.v7.4.symbols.gmt subset was downloaded from the Molecular Signatures Database to evaluate relevant pathways and molecular mechanisms. The minimum and maximum gene set sizes were set to 5 and 5000, respectively, with 1000 permutations, and a p-value < 0.05 was considered statistically significant.

### 2.5 Survival prognosis analysis

Survival data for patients were acquired from TCGA. The optimal cutoff value for the CS Ratio was calculated using the maxstat package in R, with the minimum sample size for grouping set to greater than 25% and the maximum sample size set to less than 75%. The optimal cut-off value was ultimately determined as: 0.920384. Based on this optimal cutoff, patients were categorized into High and Low groups. The survival differences between these two groups were analyzed further using the survfit function from the survival package in R, and the log-rank test was employed to assess the significance of prognosis differences among the different groups.

### 2.6 Correlation with clinical staging and independent prognostic factors

The expression differences of the CS Ratio in cervical cancer patients across various clinical T stages and overall stages were calculated using R. Non-paired Wilcoxon rank-sum tests and signed rank tests were employed for pairwise significance analysis, while the Kruskal-Wallis test was applied for multi-group sample comparisons. The median CS Ratio served as a threshold to categorize patients into CS Ratio Hi and Low groups, exploring the correlations between these groups and the patients’ clinical pathological features. We calculated the AUC using ROC analysis implemented in the pROC package. Specifically, patient follow-up times and CS Ratios were utilized to perform ROC analyses at 365, 1095, and 1825 days using the ROC function from pROC. The ci function was used to evaluate AUC and confidence intervals to derive the final AUC results.

Finally, univariable and multivariable Cox analyses were performed using clinical information obtained from the TCGA database, evaluating the significance of CS Ratio combined with relevant clinical parameters. Subsequently, based on the TCGA-CESC cohort, a nomogram was constructed via the Cox method using the R package rms, integrating survival time, survival status, and data from four features to assess their prognostic significance in the samples. The performance of these nomograms was evaluated through calibration curves and ROC curves.

## 3 Results

### 3.1 Abnormal expression of CXCL9 and SPP1 in cervical cancer patients

**Figure 1:**
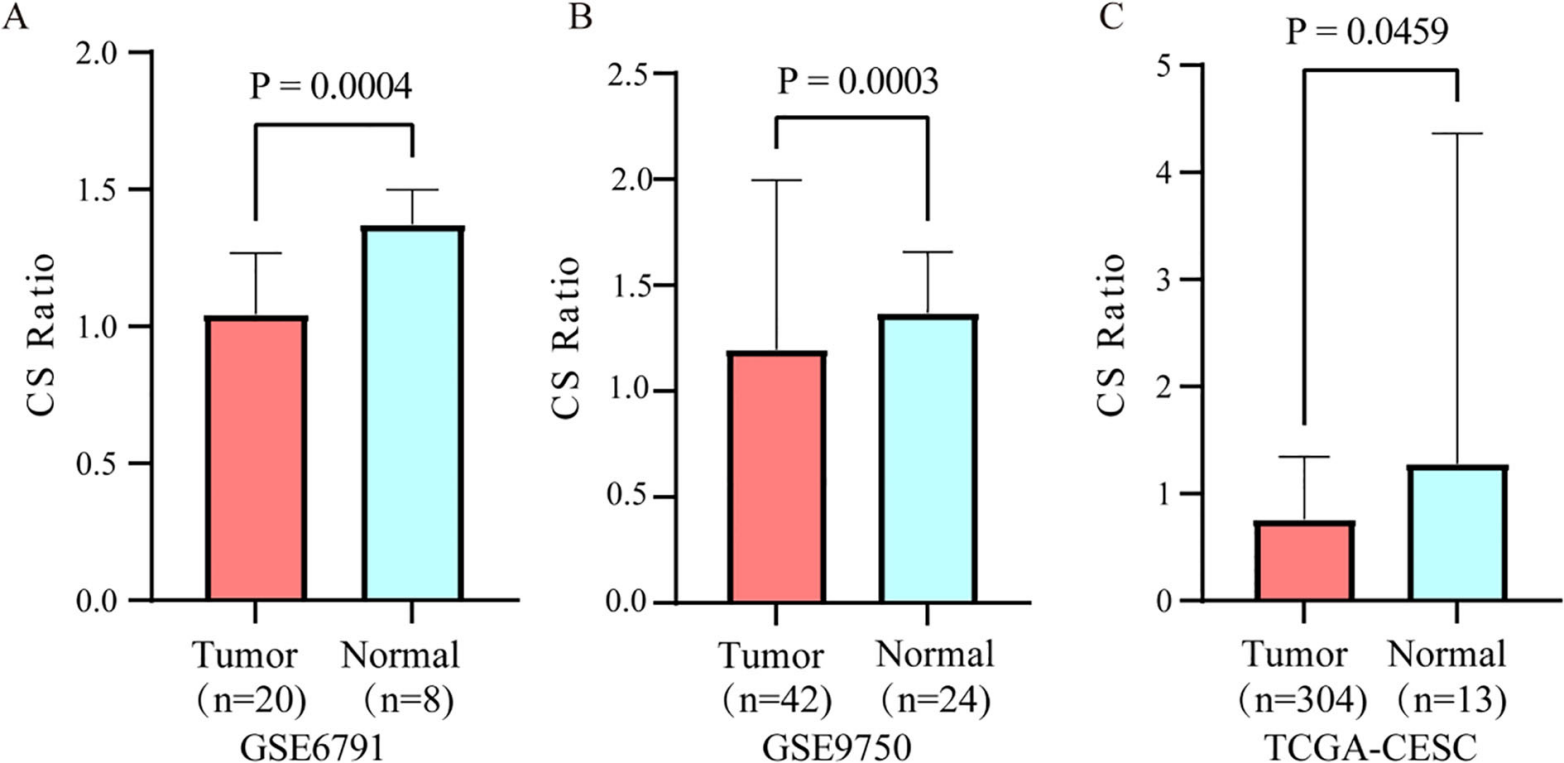
Differential expression of the CS ratio in cervical cancer and normal tissues. The CS Ratio demonstrated distinct expression patterns between cervical cancer tissues and normal cervical tissues across two independent cervical cancer patient cohorts, **(A)** GSE6791 and **(B)** GSE9750. **(C)** Differential gene expression analysis of cervical cancer tissue versus normal cervical tissue samples from the TCGA database revealed significant differences.

The CS Ratio was significantly lower in tumor tissues from cervical cancer patients compared to those from normal individuals, GSE6791 (**Figure 1A**) and GSE9750 (**Figure 1B**). Analysis of TCGA data revealed a significantly lower CS Ratio in tumor tissues from cervical cancer patients compared to normal cervical tissues (**Figure 1C**).

### 3.2 Correlation of CS ratio with the tumor immune microenvironment in cervical cancer patients

**Figure 2:**
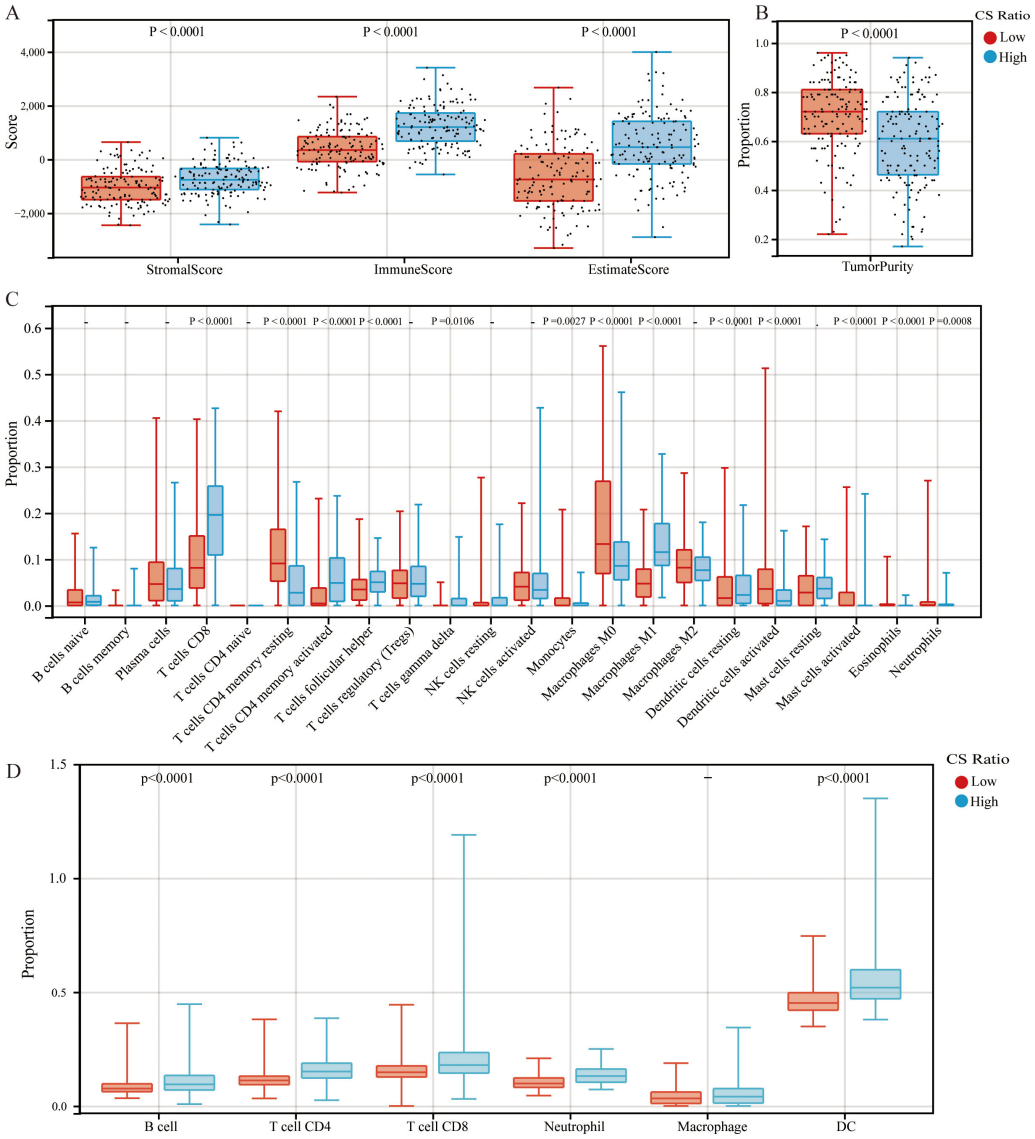
Comparisons of high and low CS Ratio groups. Cervical cancer patients were stratified into High and Low CS Ratio groups based on the median CS Ratio. Comparisons between these groups included **(A)** stromal scores, immune scores, ESTIMATE scores, **(B)** tumor purity, and **(C, D)** immune cell proportions.

Patients with cervical cancer were grouped according to CS ratio High and Low. Patients in the CS High group exhibited a higher immune score (**Figure 2A**). Tumor purity refers to the proportion of tumor cells in the TME. Previous studies have shown that tumor purity is a potential prognostic tumor indicator (25, 26). Furthermore, patients in the CS High group had lower tumor purity. This result was consistent with the immune score findings (**Figure 2B**). Patients with cervical cancer were grouped according to Tumor Purity High and Low. It could be observed that the Tumor Purity Low group demonstrated better prognosis (**Supplementary Figure 1**).

Patients with cervical cancer were grouped according to CS ratio High and Low for CIBERSORT analysis. We could observe that the CS High group had a higher proportion of CD8+ T cells and M1 macrophages, while the proportion of M2 macrophages was lower (**Figure 2C**). Furthermore, we assessed the associations between CS Ratio and immune cell infiltration levels (B cells, CD4+ T cells, CD8+ T cells, neutrophils, macrophages, and dendritic cells) using the TIMER algorithm. We could observe that in cervical cancer patients, the CS High group had a higher proportion of B cell, CD4+ T cell, CD8+ T cell, Neutrophil, and DC (**Figure 2D**). The results could be mutually validated with those from CIBERSORT.

### 3.3 The CS ratio related differential gene set and enrichment analysis

**Figure 3:**
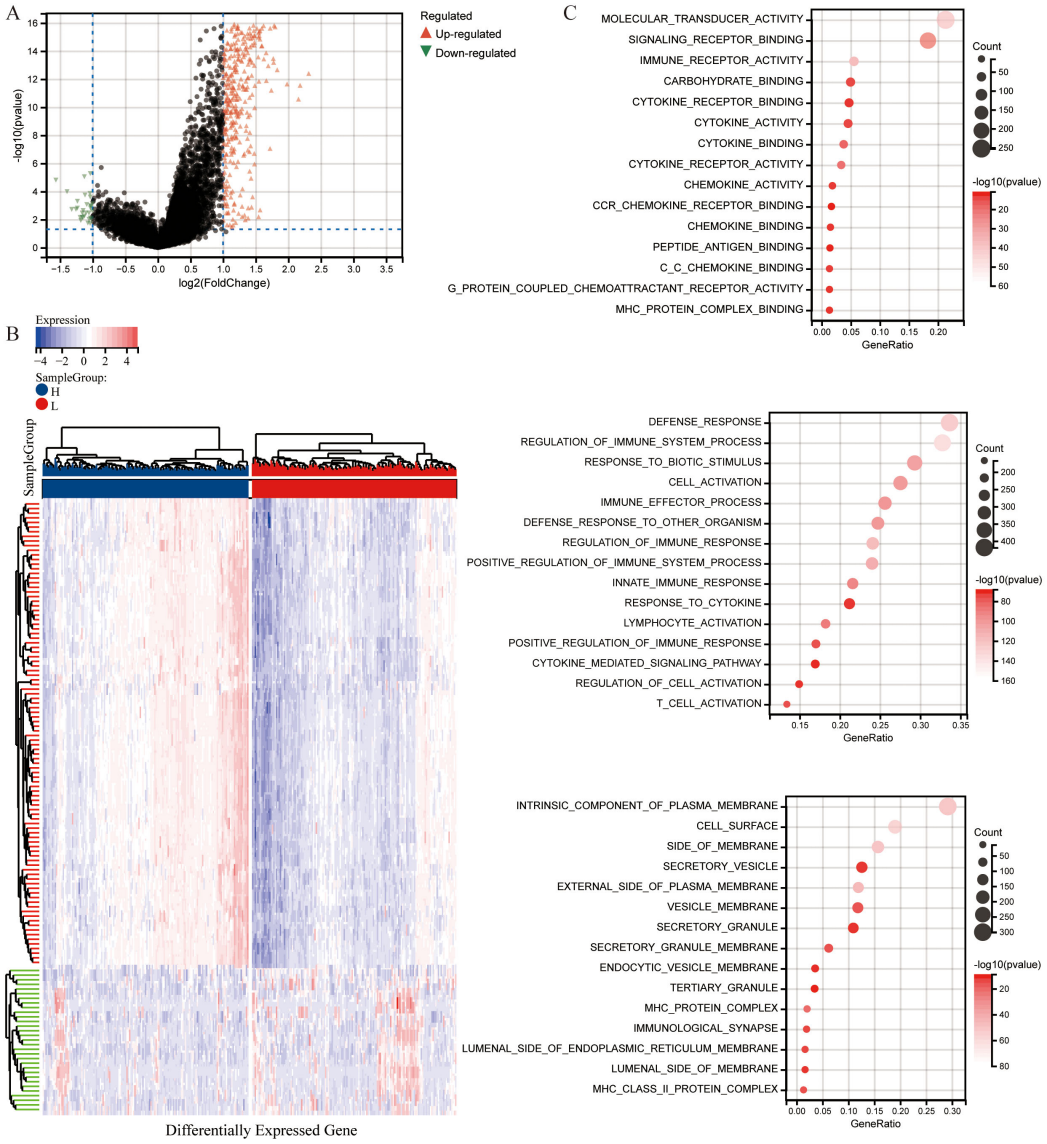
Differential analysis based on CS Ratio levels. **(A)** Patients were divided into two groups based on CS Ratio levels, and differential analysis results were visualized through a volcano plot. **(B)** A heatmap was generated to show the top 100 DEGs positively and negatively correlated with the CS Ratio. **(C)** Functional enrichment analysis of DEGs included results from Gene Ontology (GO) enrichment analysis.

To analyze significant DEGs, cervical cancer patients were stratified into High and Low groups based on the median value of the CS Ratio. The results of the significant DEGs were visualized in a volcano plot (**Figure 3A**) and a heatmap (**Figure 3B**).

The differentially expressed genes were subjected to enrichment analysis. The results of Gene Ontology (GO) analysis (**Figure 3C**). As illustrated, DEGs were predominantly enriched in immune regulation and cellular activation (lymphocytes, T cells). In terms of biological processes (GOBP), the DEGs were primarily related to immune system processes, including the regulation of immune system processes and cellular activation, as well as the modulation of immune responses. Various cellular components (GOCC) were enriched by the DEGs, including intrinsic components of the cell membrane, cell surfaces, and membrane sides. Additionally, the DEGs enriched a variety of molecular functions (GOMF), such as molecular transducer activity, signaling receptor binding, immune receptor binding, cytokine receptor binding, cytokine activity, and chemokine activity.

**Figure 4:**
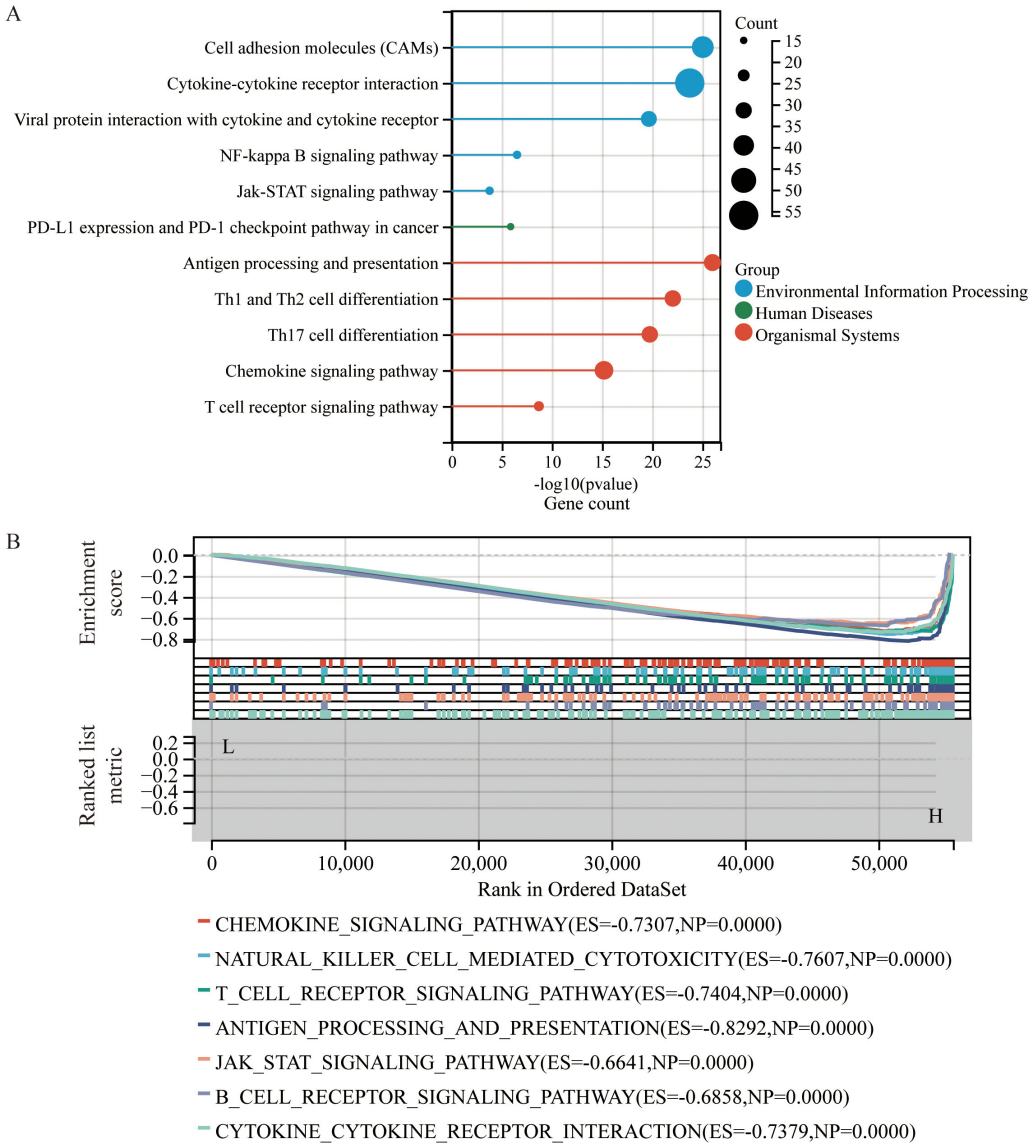
Enrichment analysis based on CS Ratio levels. **(A)** Functional enrichment analysis of DEGs included results from Kyoto Encyclopedia of Genes and Genomes (KEGG) pathway analysis. **(B)** Gene Set Enrichment Analysis (GSEA) was performed to compare the CS High subgroup with the CS Low subgroup in cervical cancer patients.

KEGG pathway enrichment analysis (**Figure 4A**) revealed that DEGs were significantly enriched in pathways related to interactions between cytokines and their receptors, interactions between viral proteins and cytokines and their receptors, Th1 and Th2 cell differentiation, the NF-κB signaling pathway, the Jak-STAT signaling pathway, PD-L1 expression and PD-1 checkpoint pathways in cancer, chemokine signaling pathways, and T cell receptor signaling pathways.

To identify potential mechanisms of the CS Ratio in cervical cancer, we performed Gene Set Enrichment Analysis (GSEA) (**Figure 4B**) using the KEGG pathway database, which revealed several signaling pathways significantly correlated with CS Ratio levels. Some of the pathways among the top 15 enrichment scores (ES) are shown in the figure. The DEGs were enriched in immune-related pathways, such as the chemokine signaling pathway (ES = -0.7307, NP = 0.0000), NK cell-mediated cytotoxicity (ES = -0.7607, NP = 0.0000), T cell receptor signaling pathway (ES = -0.7404, NP = 0.0000), antigen processing and presentation (ES = -0.8292, NP = 0.0000), Jak-STAT signaling pathway (ES = -0.6641, NP = 0.0000), B cell receptor signaling pathway (ES = -0.6858, NP = 0.0000), and interactions between cytokines and cytokine receptors (ES = -0.7379, NP = 0.0000). These findings suggest that a high CS Ratio may promote chemokine and tumor immune-related signaling pathways in cervical cancer.

### 3.4 Relationship between CS ratio and clinical pathological features and prognostic value in cervical cancer patients

**Figure 5:**
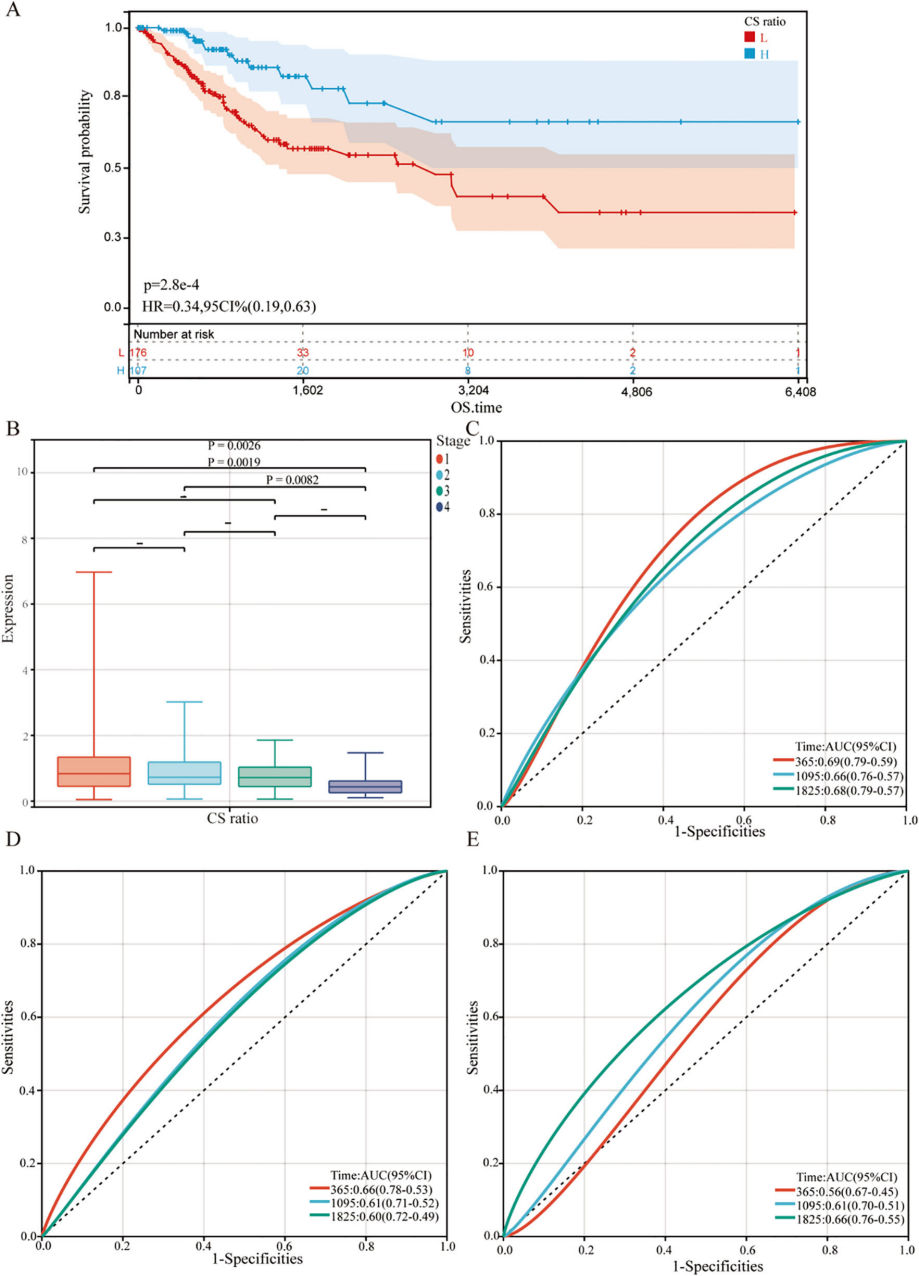
Relationship between CS Ratio and clinical pathological parameters. **(A)** Kaplan-Meier survival curves were generated to evaluate overall survival (OS) in cervical cancer patients based on the optimal cutoff value of the CS Ratio. **(B)** CS Ratio expression was also compared across different FIGO stages (Stage I to Stage IV). Time-dependent ROC curves were created to evaluate the predictive performance of a survival model incorporating the CS Ratio **(C)** and CXCL9 **(D)** and SPP1 **(E)** expression levels. The x-axis represents the false positive rate, while the y-axis represents the true positive rate.

The relationship between gene expression and overall survival (OS) in cervical cancer patients was evaluated. Kaplan-Meier analysis (**Figure 5A**) showed that using the median CS Ratio as a threshold to categorize patients into High and Low groups revealed significant prognostic differences. The hazard ratio (HR) for CS Ratio in cervical cancer patients was 0.34, indicating that a high CS Ratio is associated with better overall survival.

**Figure 6:**
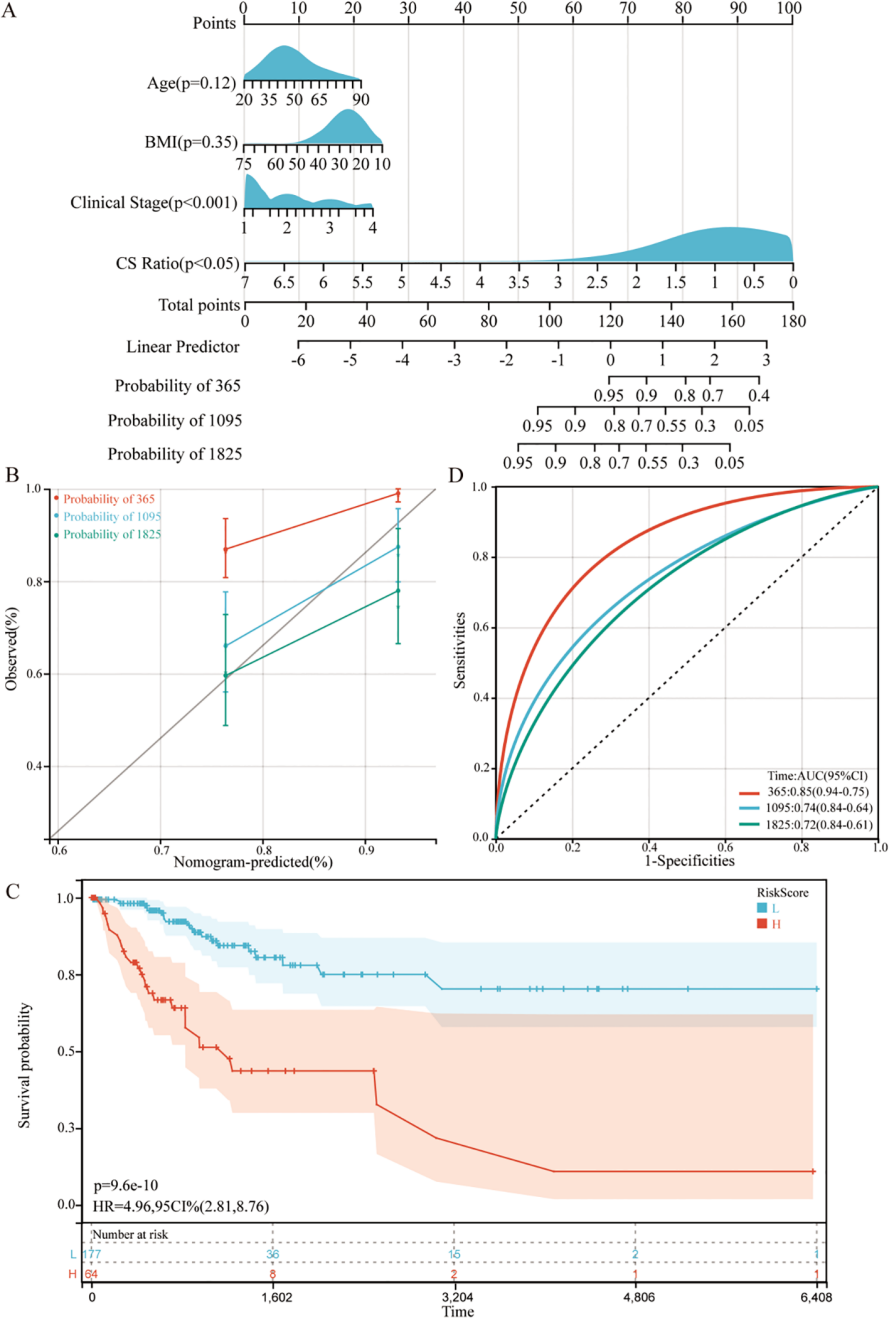
Establishment and assessment of the nomogram survival model **(A)** A nomogram was established to predict the prognosis of TCGA-CESC patients. **(B)** Kaplan–Meier analyses for the two CESC groups based on the nomogram score. **(C)** Receiver operator characteristic (ROC) analysis of the nomogram in the TCGA-CESC cohorts. **(D)** Utilization of calibration curves to verify the agreement between predicted and actual 1-year, 3-year and 5-year outcomes.

For all cervical cancer patients from TCGA, the correlation between the CS Ratio and clinicopathological characteristics of cervical cancer patients is shown (**Figure 5B**). However, neither CXCL9 nor SPP1 showed a significant correlation with Clinical Stage (**Supplementary Figure 2**). Univariate and multivariate COX regression analyses were performed to evaluate the association of CS ratio with OS in cervical cancer patients, while adjusting for clinical factors including age and clinical stage. In univariate COX analysis, a lower CS Ratio was significantly associated with poorer survival (HR=0.415, 95% CI: 0.246-0.701, P<0.001, **Table 1**). In multivariate COX analysis, even after adjusting for other confounding factors, CS Ratio maintained its independent prognostic value in CESC patients (HR=0.361, 95% CI: 0.186-0.700, P=0.003, **Table 1**). ROC analysis related to the CS Ratio demonstrated promising AUC values for predicting 1-year [AUC (95% CI) =0.69 (0.79-0.59)], 3-year [AUC (95% CI) =0.66 (0.76-0.57)], and 5-year OS [AUC (95% CI) =0.68 (0.79-0.57)] in cervical cancer patients (**Figure 5C**). Notably, these AUC values exceeded those obtained for CXCL9 (**Figure 5D**) and SPP1 (**Figure 5E**) individually. CXCL9 (**Figure 5D**) and SPP1 (**Figure 5E**) exhibit prognostic significance in cervical cancer patients. However, after adjusting for clinical factors, CXCL9 and SPP1 lost their prognostic significance (**Supplementary Figure 3**). Additionally, a prognostic model was constructed in the TCGA cohort to predict the 1-, 3-, and 5-year overall survival (OS) of CESC patients (**Figure 6A**). The overall Concordance Index of the model was 0.751, 95% CI (0.678-0.824), p=1.50x10^-11^. The calibration curve demonstrated the accurate predictive ability of this model for 1-year, 3-year, and 5-year survival rates (**Figure 6B**). Notably, significant survival differences were observed between the high-risk and low-risk groups based on the nomogram score (**Figure 6C**). To evaluate the performance of the nomogram, we accessed its predictive ability in TCGA-CESC. Our results indicated high area under the curve scores for predicting the 1-year [AUC (95% CI) =0.85 (0.94-0.75)], 3-year [AUC (95% CI) =0.74 (0.84-0.64)], and 5-year [AUC (95% CI) =0.72 (0.84-0.61)] survival of CESC patients (**Figure 6D**). These findings underscore the reliable predictive and prognostic capability of the CS Ratio.

**Table 1:** Univariate and multivariate Cox regression analyses were performed to assess the relationship between the CS Ratio and clinicopathological variables in cervical cancer patients.

Charactersitics	Total (N)	Univariate-cox analysis		Multivariate-cox analysis	
		Hazard ration (95%CI)	P value	Hazard ratio (95% CI)	P value
Age	283	1.015 (0.098-1.034)	0.092	1.02 (0.998-1.042)	0.07
Clinical stage (Stage II and Stage Ill and Stage IV vs. Stage I)	277	1.479 (0.919-2.383 )	0.107	1.725 (0.968-3.07 4)	0.064
Cervical carcinoma corpus uteri involvement indicator (PRESENT vs. ABSENT)	114	2.79 (1.037-7.491)	0.042		
Lymphovascular invasion indicator (PRESENT vs. ABSENT)	145	9.228 (2.162-39.390)	0.003		
Disease type (Non-Squamous Cell Neoplasms vs. Squamous Cell Neoplasms)	283	1.06 (0.806-1.394)	0.679		
BMI	246	0.951 (0.908-0.996)	0.032	0.967 (0.924-1.012)	0.148
CS Ratio	283	0.415 (0.246-0.701)	< 0.001	0.361 (0.186-0.700)	0.003

## 4 Discussion

Cancer mortality rates are rapidly rising worldwide and have become a leading cause of death in various countries. In this study, we are the first to analyze the relationship between the CS Ratio and cervical cancer patients, focusing on prognosis, immune scoring, clinical staging, CIBERSORT analysis, and survival prediction models.

Previous studies have established the potentially opposing roles of CXCL9 and SPP1 in cancer biology (15–17). It has been illustrated that the expression of CXCL9 and SPP1 in macrophages at the single-cell level in head and neck squamous cell carcinoma is largely mutually exclusive, with CS polarity serving as a predictive factor (22). Therefore, we investigated the correlation between CS Ratio and patient clinical factors using transcriptome sequencing data from databases. Both cervical cancer and head and neck squamous cell carcinoma are associated with a significant number of HPV infections, which are closely linked to the tumor microenvironment. Research has demonstrated that HPV-positive head and neck squamous cell carcinoma patients respond more favorably to treatment and experience improved prognoses. Thus, for cervical cancer patients, who have a stronger association with HPV, the CS Ratio may also serve as a predictive indicator and an independent prognostic factor.

Accumulating evidence indicates that infiltration of immune effector cells (particularly CD8+ T cells, NK cells, and DC cells) into TME can enhance therapeutic responses to ICIs (27–29). We are the first to demonstrate the correlation between CS Ratio and immune cells, as well as immune scores in cervical cancer patients, and to investigate the prognostic implications for patients stratified by CS Ratio. Additionally, ROC curve analysis indicated that the CS Ratio possesses greater long-term predictive capability than CXCL9 or SPP1 (1/3/5-year AUC = 0.68, 0.66, 0.67). These results suggest that the CS Ratio may serve as a more reliable prognostic biomarker for cervical cancer patients. Our study establishes a foundation for a detailed investigation of the relationship between CS Ratio and the tumor-associated immune microenvironment.

In our study, we first demonstrated that patients with a high CS Ratio in cervical cancer exhibit better prognoses, positively correlating with CD8+ T cells and M1 macrophages, with a correspondingly lower proportion of M2 macrophages. Comparisons in immune scores indicated that patients in the CS Ratio High group had higher immune scores and lower tumor purity. CIBERSORT analysis revealed a higher proportion of CD8+ T cells and M1 macrophages in the CS Ratio High group, whereas M2 macrophages showed a significantly lower proportion. Moreover, our analysis indicated a significant decrease in CS Ratio with advancing Stage and T stage. Univariate and multivariate Cox analyses affirmed that CS Ratio could serve as an independent prognostic factor.

In summary, our findings formally establish the role of the CS Ratio in cervical cancer patients, indicating that a high CS Ratio enhances anti-tumor immunity and is associated with significantly extended survival. These results emphasize the potential key role of the CS Ratio in macrophage classification and immune cell infiltration. However, the specific molecular mechanisms underlying the antitumor effects of CS Ratio in cervical cancer patients and its exact functional roles require further exploration and validation through both *in vitro* and *in vivo* experiments. In summary, CS Ratio may function as a more effective prognostic and predictive indicator for cervical cancer patients.

## Data Availability

Publicly available datasets were analyzed in this study. This data can be found here: https://www.genome.gov/Funded-Programs-Projects/Cancer-Genome-Atlas & https://www.ncbi.nlm.nih.gov/geo/.

## References

[1] Bray F Ferlay J Soerjomataram I Siegel RL Torre LA Jemal A. Erratum: Global cancer statistics 2018: GLOBOCAN estimates of incidence and mortality worldwide for 36 cancers in 185 countries. CA Cancer J Clin. (2020) 70:313. doi: 10.3322/caac.21609, PMID: 30207593

[2] Revathidevi S Murugan AK Nakaoka H Inoue I Munirajan AK. APOBEC: A molecular driver in cervical cancer pathogenesis. Cancer Lett. (2021) 496:104–16. doi: 10.1016/j.canlet.2020.10.004, PMID: 33038491 PMC7539941

[3] Bhat AA Nisar S Maacha S Carneiro-Lobo TC Akhtar S Siveen KS. Cytokine-chemokine network driven metastasis in esophageal cancer; promising avenue for targeted therapy. Mol Cancer. (2021) 20:2. doi: 10.1186/s12943-020-01294-3, PMID: 33390169 PMC7780621

[4] Bule P Aguiar SI Aires-Da-Silva F Dias JNR. Chemokine-directed tumor microenvironment modulation in cancer immunotherapy. Int J Mol Sci. (2021) 22:9804. doi: 10.3390/ijms22189804, PMID: 34575965 PMC8464715

[5] Karin N. CXCR3 ligands in cancer and autoimmunity, chemoattraction of effector T cells, and beyond. Front Immunol. (2020) 11:976. doi: 10.3389/fimmu.2020.00976, PMID: 32547545 PMC7274023

[6] Chow MT Ozga AJ Servis RL Frederick DT Lo JA Fisher DE. Intratumoral activity of the CXCR3 chemokine system is required for the efficacy of anti-PD-1 therapy. Immunity. (2019) 50:1498–512.e5. doi: 10.1016/j.immuni.2019.04.010, PMID: 31097342 PMC6527362

[7] Bronger H Singer J Windmüller C Reuning U Zech D Delbridge C. CXCL9 and CXCL10 predict survival and are regulated by cyclooxygenase inhibition in advanced serous ovarian cancer. Br J Cancer. (2016) 115:553–63. doi: 10.1038/bjc.2016.172, PMID: 27490802 PMC4997538

[8] Bonfill-Teixidor E Neva-Alejo A Arias A Cuartas I Iurlaro R Planas-Rigol E. Cervical cancer evades the host immune system through the inhibition of type I interferon and CXCL9 by LIF. Clin Cancer research: an Off J Am Assoc Cancer Res. (2024) 30:4505–16. doi: 10.1158/1078-0432.Ccr-24-0385, PMID: 39078728

[9] Su X Xu BH Zhou DL Ye ZL He HC Yang XH. Polymorphisms in matricellular SPP1 and SPARC contribute to susceptibility to papillary thyroid cancer. Genomics. (2020) 112:4959–67. doi: 10.1016/j.ygeno.2020.09.018, PMID: 32919020

[10] Zeng B Zhou M Wu H Xiong Z. SPP1 promotes ovarian cancer progression via Integrin β1/FAK/AKT signaling pathway. OncoTargets Ther. (2018) 11:1333–43. doi: 10.2147/ott.S154215, PMID: 29559792 PMC5856063

[11] Kijewska M Kocyk M Kloss M Stepniak K Korwek Z Polakowska R. The embryonic type of SPP1 transcriptional regulation is re-activated in glioblastoma. Oncotarget. (2017) 8:16340–55. doi: 10.18632/oncotarget.14092, PMID: 28030801 PMC5369967

[12] Lin Y Weng Z Zhang F Chong Y. SPP1 could serve as a prognostic biomarker for patients with hepatocellular carcinoma. Asian J Surg. (2025) 48:2997–9. doi: 10.1016/j.asjsur.2025.01.086

[13] Song SZ Lin S Liu JN Zhang MB Du YT Zhang DD. Targeting of SPP1 by microRNA-340 inhibits gastric cancer cell epithelial-mesenchymal transition through inhibition of the PI3K/AKT signaling pathway. J Cell Physiol. (2019) 234:18587–601. doi: 10.1002/jcp.28497, PMID: 30953349

[14] Zhao K Ma Z Zhang W. Comprehensive analysis to identify SPP1 as a prognostic biomarker in cervical cancer. Front Genet. (2021) 12:732822. doi: 10.3389/fgene.2021.732822, PMID: 35058964 PMC8764398

[15] Tokunaga R Zhang W Naseem M Puccini A Berger MD Soni S. CXCL9, CXCL10, CXCL11/CXCR3 axis for immune activation - A target for novel cancer therapy. Cancer Treat Rev. (2018) 63:40–7. doi: 10.1016/j.ctrv.2017.11.007, PMID: 29207310 PMC5801162

[16] McAllister SS Gifford AM Greiner AL Kelleher SP Saelzler MP Ince TA. Systemic endocrine instigation of indolent tumor growth requires osteopontin. Cell. (2008) 133:994–1005. doi: 10.1016/j.cell.2008.04.045, PMID: 18555776 PMC4121664

[17] Dangaj D Bruand M Grimm AJ Ronet C Barras D Duttagupta PA. Cooperation between constitutive and inducible chemokines enables T cell engraftment and immune attack in solid tumors. Cancer Cell. (2019) 35:885–900.e10. doi: 10.1016/j.ccell.2019.05.004, PMID: 31185212 PMC6961655

[18] Anfray C Ummarino A Andón FT Allavena P. Current strategies to target tumor-associated-macrophages to improve anti-tumor immune responses. Cells. (2019) 9:46. doi: 10.3390/cells9010046, PMID: 31878087 PMC7017001

[19] Choi Y Lee D Kim NY Seo I Park NJ Chong GO. Role of tumor-associated macrophages in cervical cancer: integrating classical perspectives with recent technological advances. Life (Basel Switzerland). (2024) 14:443. doi: 10.3390/life14040443, PMID: 38672714 PMC11051155

[20] Yunna C Mengru H Lei W Weidong C. Macrophage M1/M2 polarization. Eur J Pharmacol. (2020) 877:173090. doi: 10.1016/j.ejphar.2020.173090, PMID: 32234529

[21] Fridman WH Zitvogel L Sautès-Fridman C Kroemer G. The immune contexture in cancer prognosis and treatment. Nat Rev Clin Oncol. (2017) 14:717–34. doi: 10.1038/nrclinonc.2017.101, PMID: 28741618

[22] Bill R Wirapati P Messemaker M Roh W Zitti B Duval F. CXCL9:SPP1 macrophage polarity identifies a network of cellular programs that control human cancers. Sci (New York NY). (2023) 381:515–24. doi: 10.1126/science.ade2292, PMID: 37535729 PMC10755760

[23] Shaikh MH Bortnik V McMillan NA Idris A. cGAS-STING responses are dampened in high-risk HPV type 16 positive head and neck squamous cell carcinoma cells. Microbial pathogenesis. (2019) 132:162–5. doi: 10.1016/j.micpath.2019.05.004, PMID: 31054871

[24] Li S Hong X Wei Z Xie M Li W Liu G. Ubiquitination of the HPV oncoprotein E6 is critical for E6/E6AP-mediated p53 degradation. Front Microbiol. (2019) 10:2483. doi: 10.3389/fmicb.2019.02483, PMID: 31749782 PMC6842930

[25] Gong Z Zhang J Guo W. Tumor purity as a prognosis and immunotherapy relevant feature in gastric cancer. Cancer Med. (2020) 9:9052–63. doi: 10.1002/cam4.3505, PMID: 33030278 PMC7724479

[26] Mao Y Feng Q Zheng P Yang L Liu T Xu Y. Low tumor purity is associated with poor prognosis, heavy mutation burden, and intense immune phenotype in colon cancer. Cancer Manage Res. (2018) 10:3569–77. doi: 10.2147/cmar.S171855, PMID: 30271205 PMC6149864

[27] Shimasaki N Jain A Campana D. NK cells for cancer immunotherapy. Nat Rev Drug Discov. (2020) 19:200–18. doi: 10.1038/s41573-019-0052-1, PMID: 31907401

[28] Sabado RL Balan S Bhardwaj N. Dendritic cell-based immunotherapy. Cell Res. (2017) 27:74–95. doi: 10.1038/cr.2016.157, PMID: 28025976 PMC5223236

[29] Tumeh PC Harview CL Yearley JH Shintaku IP Taylor EJ Robert L. PD-1 blockade induces responses by inhibiting adaptive immune resistance. Nature. (2014) 515:568–71. doi: 10.1038/nature13954, PMID: 25428505 PMC4246418

