# Correction: CS Ratio is an immune-related prognostic biomarker for cervical cancer

**DOI:** 10.3389/fonc.2025.1697346

**Published:** 2025-09-12

**Authors:** Peiqin Shi, Wenwen Zhang, Qingqing Yao, Zhanna Yuan, Jiaqi Wang, Ziye Yang, Pengpeng Qu

**Affiliations:** ^1^ Clinical School of Obstetrics and Gynecology Center, Tianjin Medical University, Tianjin, China; ^2^ Tianjin Institute of Gynecology Obstetrics, Tianjin Central Hospital of Gynecology Obstetrics, Tianjin, China; ^3^ Department of Gynecological Oncology, Tianjin Central Hospital of Gynecology Obstetrics, School of Medicine, Nankai University, Tianjin, China; ^4^ Department of Obstetrics and Gynecology, Tianjin Medical University Baodi Hospital, Tianjin, China; ^5^ Department of Gynecological Oncology, Tianjin Central Hospital of Gynecology Obstetrics, Tianjin, China

**Keywords:** SPP1, CXCL9, cervical cancer, TCGA, GEO

Authors “Peiqin Shi” and “Wenwen Zhang” were erroneously omitted as equal contributing author.

The original version of this article has been updated.

