# Supplementary figures and images for: CS Ratio is an immune-related prognostic biomarker for cervical cancer
Source: Front Oncol. 2025 Aug 27;15:1547529. doi: 10.3389/fonc.2025.1547529 (PMC12420272; doi:10.3389/fonc.2025.1547529)

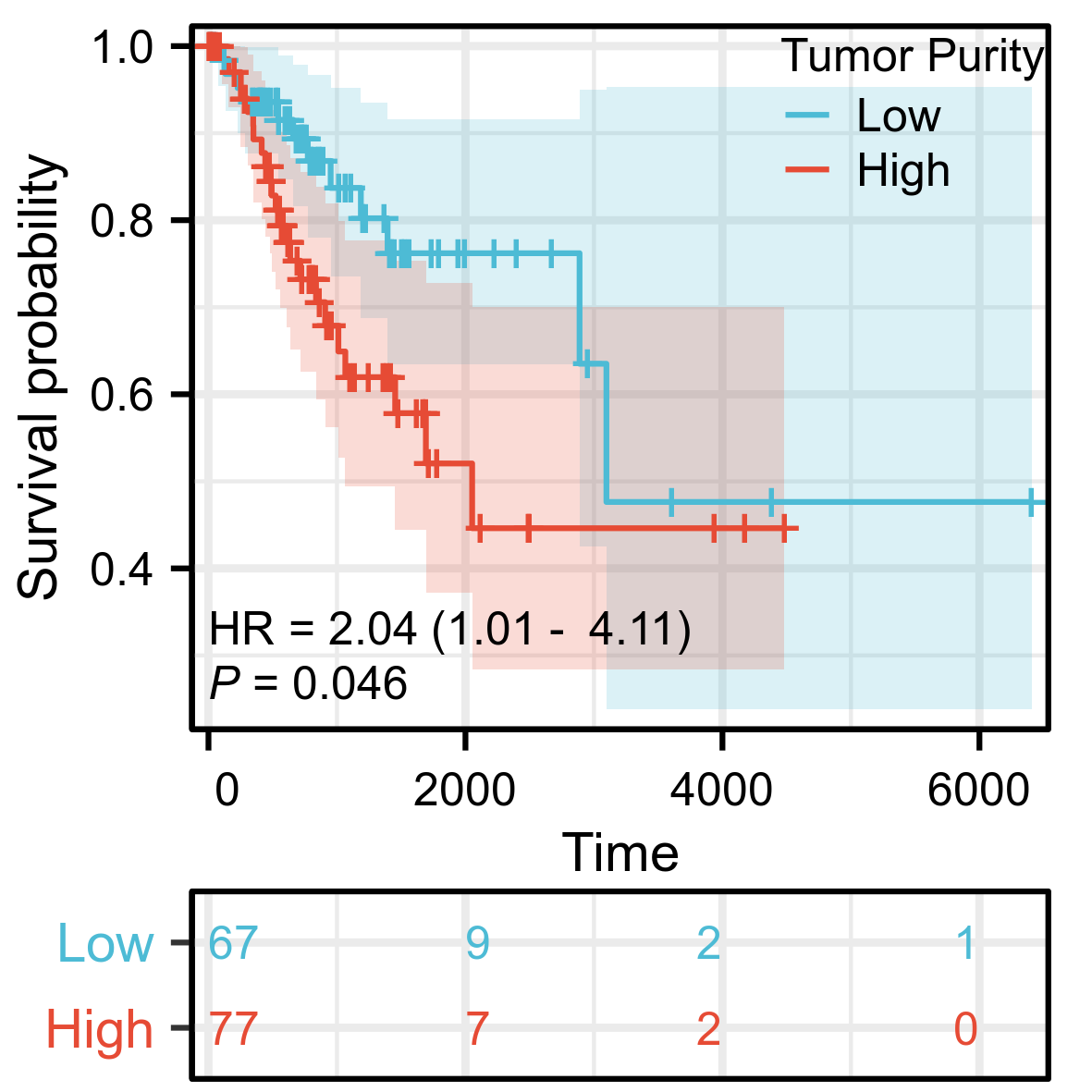

Supplement: Supplementary file 1 [file Image1.tiff]

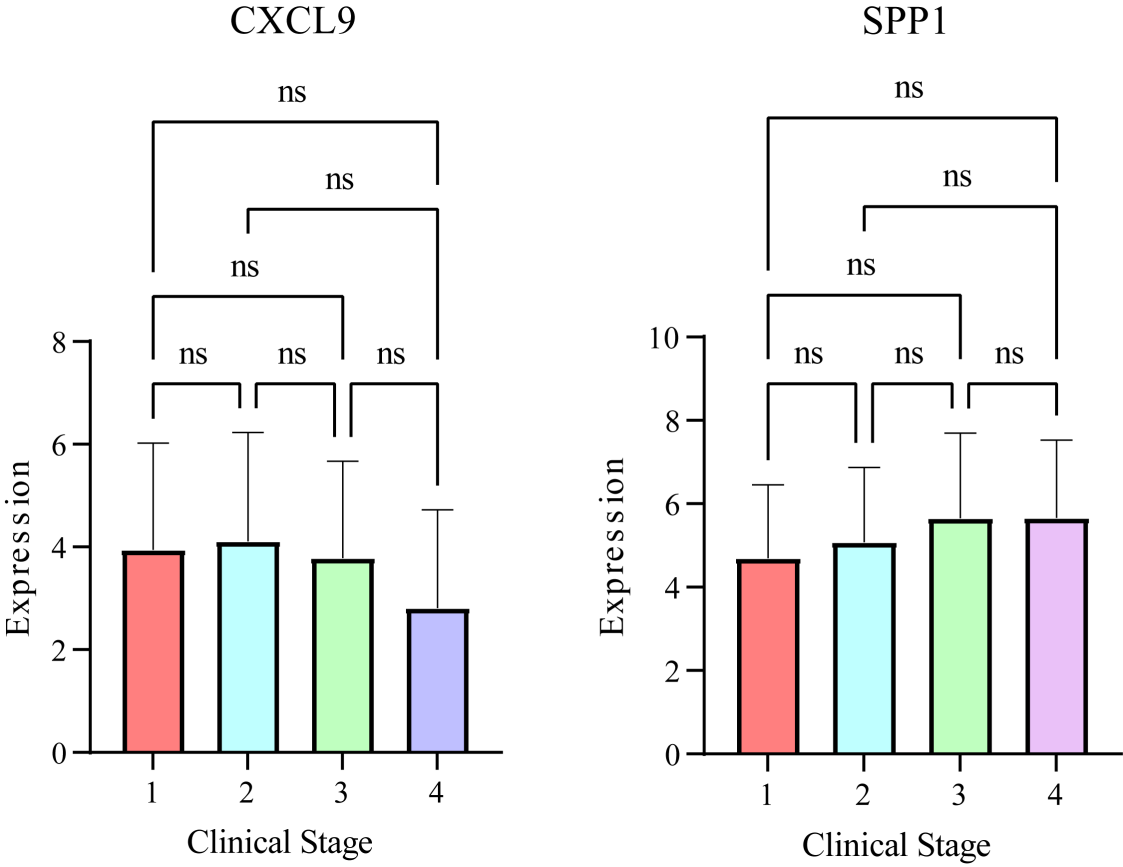

Supplement: Supplementary file 2 [file Image2.tiff]

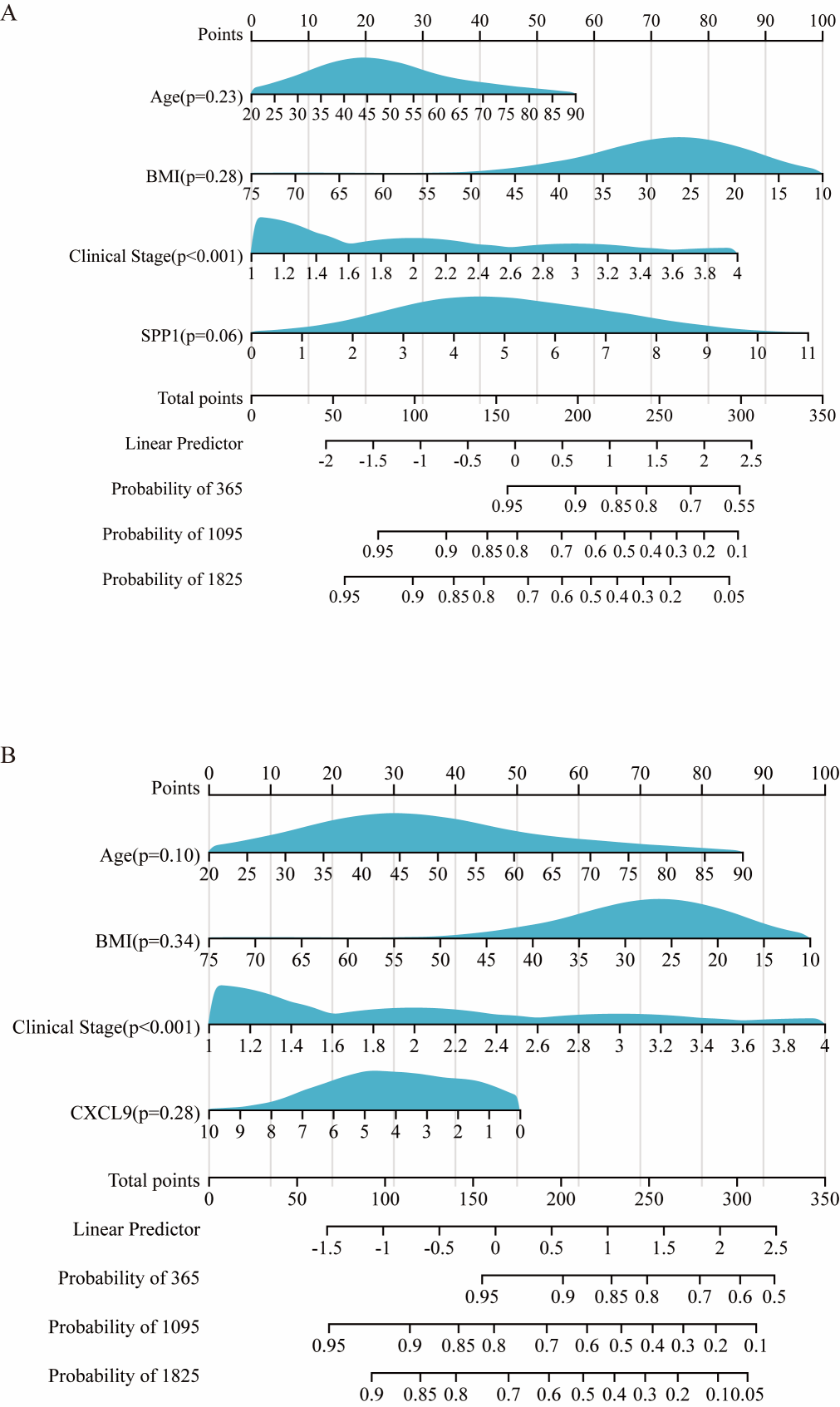

Supplement: Supplementary file 3 [file Image3.tiff]
